# Supplementary material for: Defining a core configuration for human centromeres during mitosis
Source: Nat Commun. 2023 Dec 1;14:7947. doi: 10.1038/s41467-023-42980-2 (PMC10692335; doi:10.1038/s41467-023-42980-2)
Supplement: Supplementary file 1 — Supplementary Information [file 41467_2023_42980_MOESM1_ESM.pdf]

Figure S1

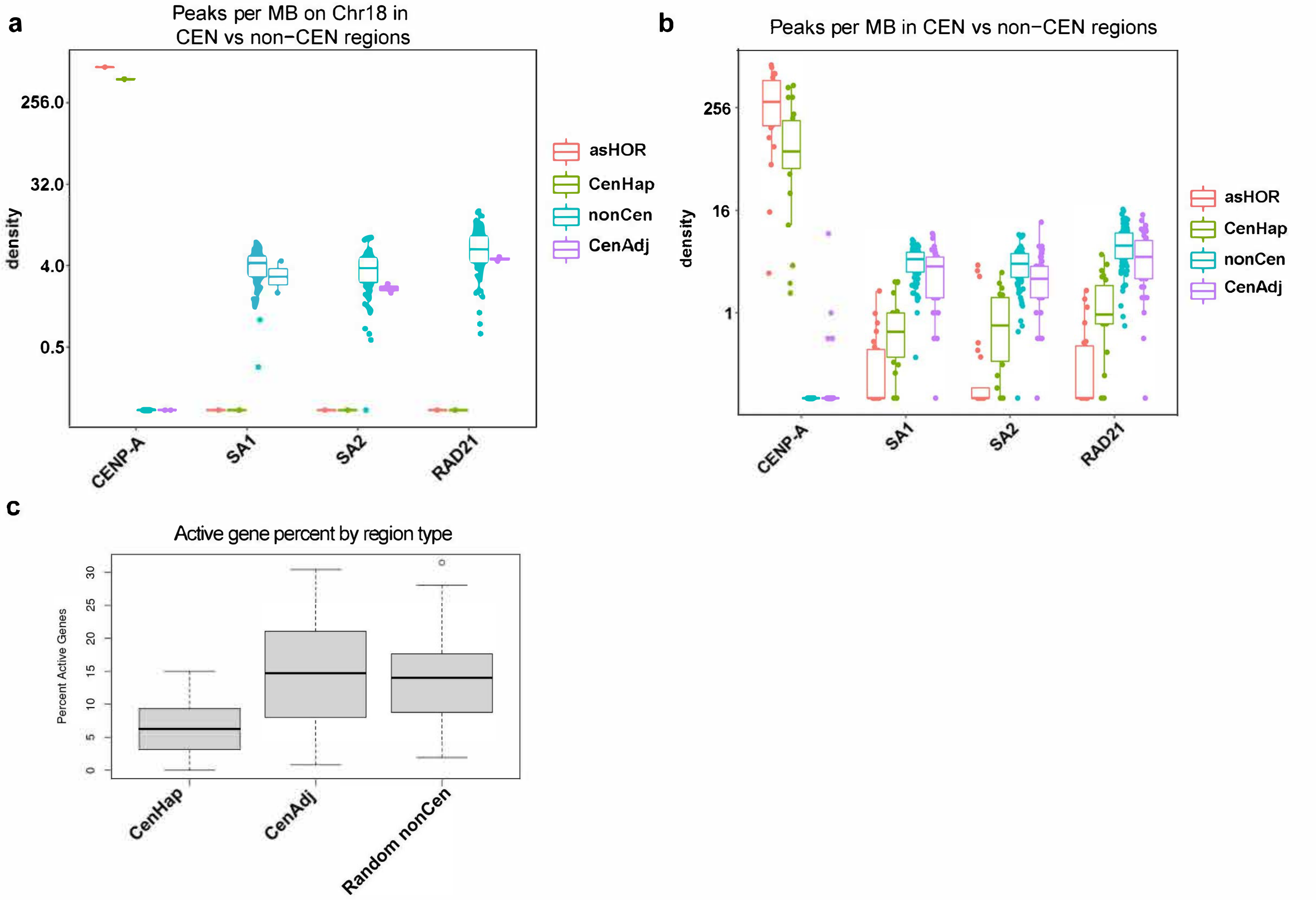

Figure S1. Cohesin enrichment at human pericentromeres. Peaks were called after mapping ChIP-seq data for RAD21, SA1 and SA2 from RPE-1 cells to the CHM13 v1.0 reference genome. Common peaks from N=2 biological replicates of RPE-1 were merged to generate a reference set of peaks. **a.** Peak densities (number of peaks per mb) of CENP-A, SA1, SA2 and RAD21 within active α-satellite HOR (asHOR), centromere haplotype (CenHap), 2 mb regions flanking CenHap (CenAdj) and 100 random genomic regions outside of the CenHap (nonCen) of chromosome 18 in RPE1 cells. **b.** Metagenome analysis of peak densities (log2) of CENP-A, SA1, SA2 and RAD21 within the above stated regions, across all chromosomes. **c.** Number of active genes within the centromere haplotypes (CenHap) and 2 Mb flanking the CenHap (CenAdj) for all chromosomes, and (100 random regions in the genome, Random nonCen) was determined from previously published RNAseq data from CHM13 cells. See Supplementary Table S1 for detailed numbers. Plot shows percent of active genes for every region. For all box plots, upper and lower whiskers represent the largest and smallest values no further than 1.5 * inter quartile range (IQR) of the bounds, respectively. Data outside of whiskers are outlying points. Center denotes median, and lower and upper bounds of box denote 25th and 75th percentiles, respectively.

Figure S2

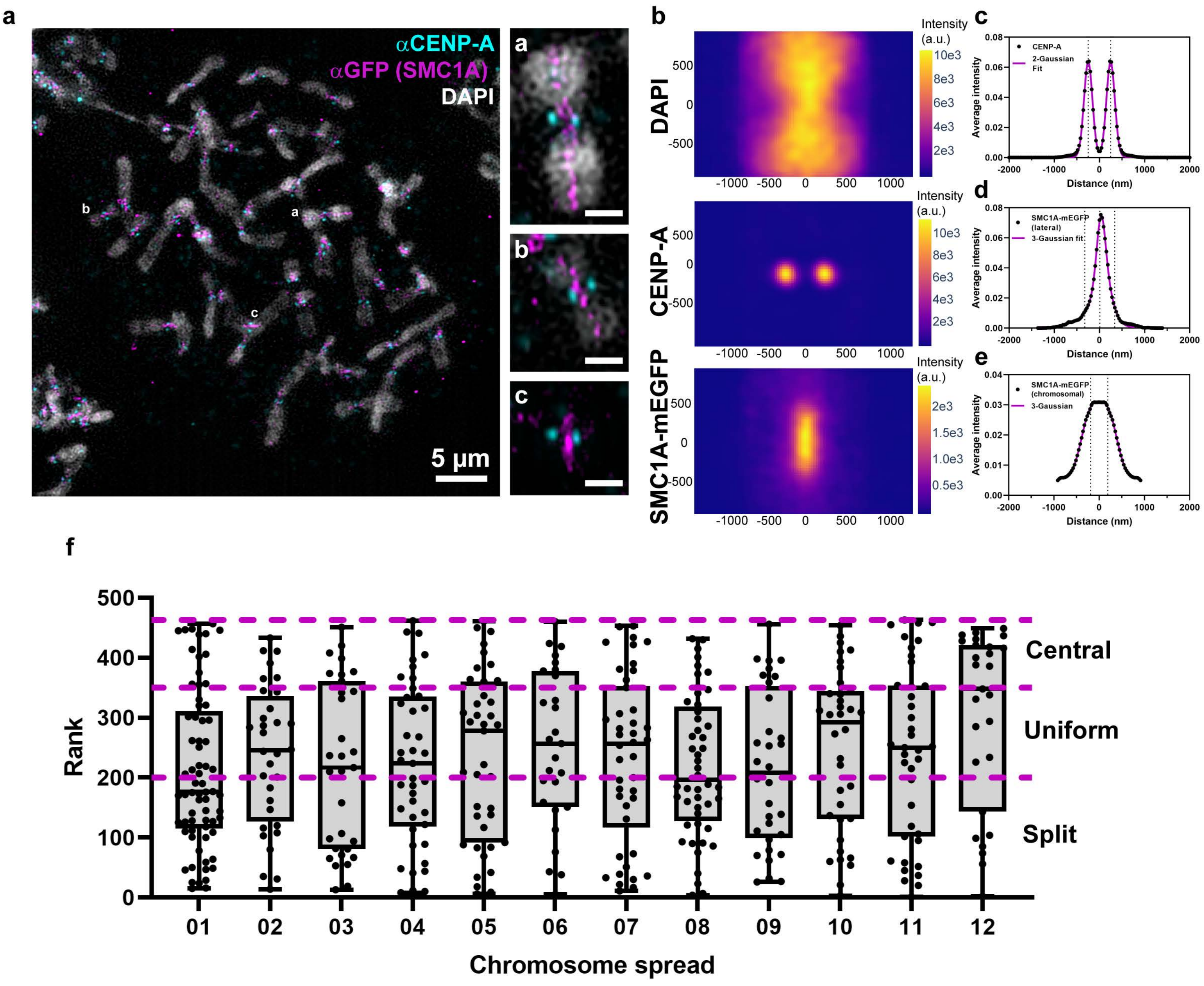

Fig. S2. Single particle averaging of 3D-SIM images with aligned CENP-A and cohesin signals.
a. SIM images of native mitotic chromosomes display localization of centromere components CENP-A (cyan) and SMC1A-mEGFP visualized by immunostaining with anti-GFP antibody (magenta). Zoomed images of three individual centromeres show CENP-A and SMC1A-mEGFP. **b.** Summed intensities of DAPI, CENP-A and SMC1A-mEGFP (probed with anti-GFP) around centromeres. Data representative of n=319 chromosomes (12 spreads) from one biological replicate. x and y axes indicate distance (nm). **c-e.** Intensities of CENP-A measured along the lateral axis and SMC1A measured along the lateral and chromosomal axes (black closed circled), along with their Gaussian fits (magenta line). Dotted line represent positions of peaks from the fits. **f.** Breakdown of RAD21 classes across multiple chromosome spreads show a range of RAD21 conformations per spread. Data representative of n=463 chromosomes from 12 mitotic spreads from one biological replicate were assigned a rank based on their center-enriched RAD21 ratio (See Figure 4a). Dots denote assigned ranks for each chromosome. Box and whisker plot showing the distribution of assigned ranks of chromosomes from every mitotic spread. Chromosomes with all ranks (diverse center-enriched RAD21 ratio) are uniformly distributed across all mitotic spreads, denoting that no particular class of RAD21 (split, uniform or central) is exclusively contributed by certain spreads. For box plot, minima and maxima denote 0 and 100th percentile, respectively, center denotes median, and lower and upper bounds of box denote 25th and 75th percentile of assigned ranks, respectively.

Figure S3

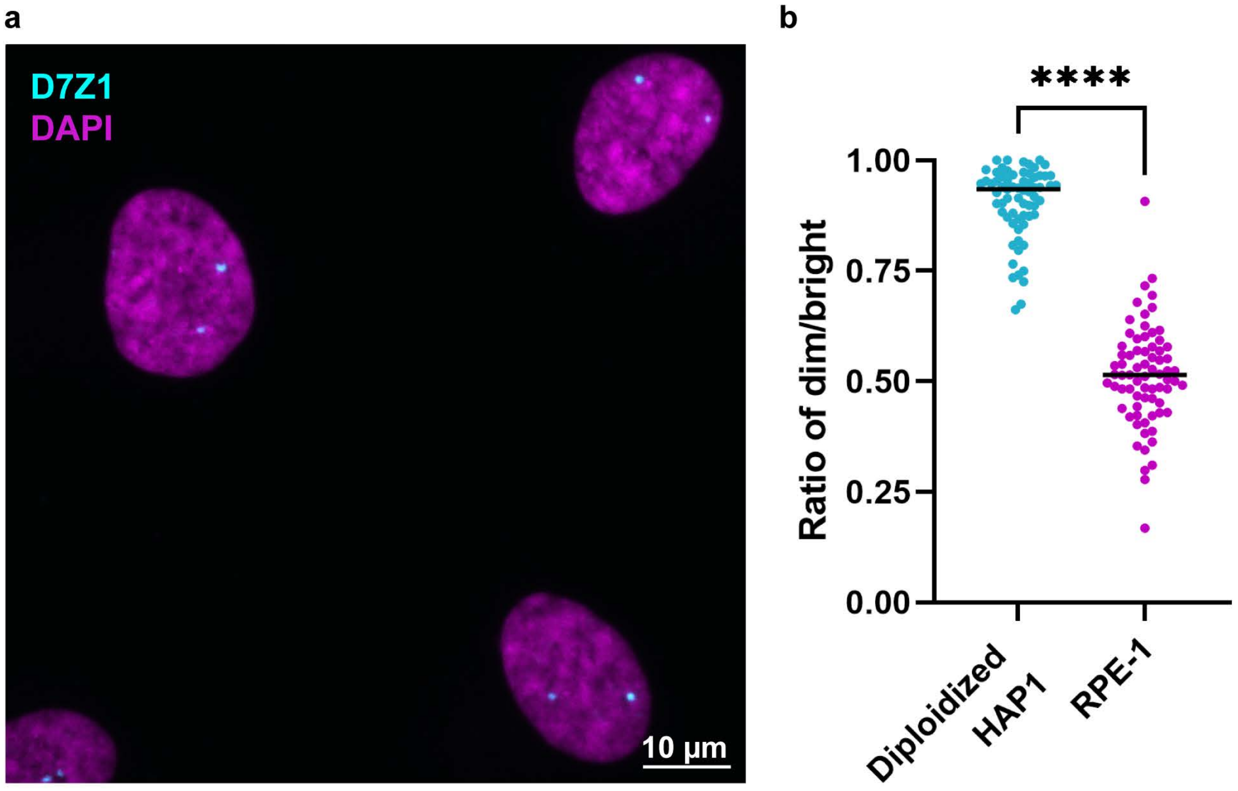

Fig. S3. RPE-1 cells display copy number variation at the D7Z1 HOR array on the two homologs of chromosome 7. **a.** FISH performed with a probe to D7Z1 on G2-arrested RPE-1 cells shows two foci (cyan) in each nucleus (DAPI, magenta). One homolog is lower in intensity than the other. **b.** Intensities of the pair of D7Z1 signals within each nucleus were determined from diploidized HAP-1 cells (homozygous chromosome 7, control) and RPE-1 cells. The plot shows the ratio of the lower intensity value over the higher intensity value for each pair of signals per nucleus. Diploidized HAP-1 and RPE-1 cells show a median ratio of 0.93 and 0.51, respectively. Medians were compared by one-way Mann-Whitney test, ****$p<0.0001$.

Table S1. Number of cohesin binding sites in upstream and downstream regions of genes within CenHap and non-CenHap regions[&&]

Upstream and Downstream Gene Regions

Gene and overlap counts by factor

	Gene state		SA1		SA2		RAD21		CTCF	
	active	inactive	active	inactive	active	inactive	active	inactive	active	inactive
cenUp	228	3873	18 (7.89%)	65 (1.67%)	20 (8.77%)	38 (0.98%)	45 (19.74%)	94 (2.43%)	16 (7.02%)	65 (1.68%)
cenDn	229	3874	9 (3.93%)	49 (1.26%)	10 (4.37%)	35 (0.90%)	19 (8.30%)	71 (1.83%)	7 (3.06%)	65 (1.68%)
nonCenUp	9874	46112	756 (7.68%)	1124 (2.44%)	762 (7.74%)	1222 (2.65%)	2080 (21.12%)	2722 (5.90%)	589 (5.98%)	1363 (2.96%)
nonCenDn	9848	46113	487 (4.95%)	954 (2.07%)	545 (5.53%)	1015 (2.20%)	1340 (13.61%)	2198 (4.75%)	376 (3.82%)	1109 (2.40%)

[&&]Table shows overlap of cohesin binding sites with respective genic regions (500 bp region 1000 bp upstream or downstream of TSS). cenUp – Promoter upstream regions of active/inactive centromeric genes. cenDn – Promoter downstream regions of active/inactive centromeric genes. nonCenUp – Promoter upstream regions of active or inactive non-centromeric genes. nonCenDn – Promoter downstream regions of active or inactive non-centromeric genes. As an example, cenUp-SA1-active denotes the number of SA1 peaks overlapping with the promoter upstream region of active centromeric genes. Percent of these numbers are denoted in brackets. As an example, 7.89% of all SA1 peaks overlap with the promoter upstream region of active centromeric genes.
