# Supplementary material for: Defining a core configuration for human centromeres during mitosis
Source: Nat Commun. 2023 Dec 1;14:7947. doi: 10.1038/s41467-023-42980-2 (PMC10692335; doi:10.1038/s41467-023-42980-2)
Supplement: Supplementary file 3 — Reporting Summary [file 41467_2023_42980_MOESM3_ESM.pdf]

nature portfolio

Corresponding author(s): Jennifer Gerton

Last updated by author(s): 2023/10/16

Reporting Summary

Nature Portfolio wishes to improve the reproducibility of the work that we publish. This form provides structure for consistency and transparency in reporting. For further information on Nature Portfolio policies, see our Editorial Policies and the Editorial Policy Checklist.

Statistics

For all statistical analyses, confirm that the following items are present in the figure legend, table legend, main text, or Methods section.

n/a	Confirmed	
☐	☒	The exact sample size (n) for each experimental group/condition, given as a discrete number and unit of measurement
☐	☒	A statement on whether measurements were taken from distinct samples or whether the same sample was measured repeatedly
☐	☒	The statistical test(s) used AND whether they are one- or two-sided<br>*Only common tests should be described solely by name; describe more complex techniques in the Methods section.*
☒	☐	A description of all covariates tested
☐	☒	A description of any assumptions or corrections, such as tests of normality and adjustment for multiple comparisons
☐	☒	A full description of the statistical parameters including central tendency (e.g. means) or other basic estimates (e.g. regression coefficient) AND variation (e.g. standard deviation) or associated estimates of uncertainty (e.g. confidence intervals)
☐	☒	For null hypothesis testing, the test statistic (e.g. F, t, r) with confidence intervals, effect sizes, degrees of freedom and P value noted<br>*Give P values as exact values whenever suitable.*
☒	☐	For Bayesian analysis, information on the choice of priors and Markov chain Monte Carlo settings
☒	☐	For hierarchical and complex designs, identification of the appropriate level for tests and full reporting of outcomes
☒	☐	Estimates of effect sizes (e.g. Cohen's d, Pearson's r), indicating how they were calculated

Our web collection on statistics for biologists contains articles on many of the points above.

Software and code

Policy information about availability of computer code

Data collection	3D-SIM images were acquired using Applied Precision OMX Blaze V4 structured illumination microscope (GE Healthcare) and Elyra 7 microscope (Carl Zeiss AG) using the companies provided softwares - DeltaVision OMX v3.70 and ZEN 3.0 SR FP2, respectively. Confocal images were acquired using Zen software (black edition for LSM-780, version 14.0.24.201) on a Zeiss LSM780 microscope.
Data analysis	ChIP-seq reads were demultiplexed using Illumina Real Time Analysis version RTA 2.4.11 and bcl2fastq2 v2.20. ChIP-seq data was analyzed using bwa version 0.7.17-r1188, bowtie2 version 2.4.2, samtools version 1.14, GenomicRanges package version 1.48.0, macs version 2.1.2. RNA-seq data was aligned using STAR version 2.7.3a. 3D-SIM images were reconstructed using SoftWoRx software v.6.5.2 (GE Healthcare). 3D-SIM images obtained after reconstruction were analyzed using several custom open-source FIJI analysis packages available at http:// research.stowers.org/ imagejplugins/ and https://github.com/jouyun/2023_Gupta. Statistical analysis was performed using R v4.2.0 or GraphPad Prism v 9.0. Images were processed using Fiji vl.54e and Adobe Illustrator 26.3.l.

For manuscripts utilizing custom algorithms or software that are central to the research but not yet described in published literature, software must be made available to editors and reviewers. We strongly encourage code deposition in a community repository (e.g. GitHub). See the Nature Portfolio guidelines for submitting code & software for further information.

Data

Policy information about availability of data

All manuscripts must include a data availability statement. This statement should provide the following information, where applicable:

- Accession codes, unique identifiers, or web links for publicly available datasets
- A description of any restrictions on data availability
- For clinical datasets or third party data, please ensure that the statement adheres to our policy

The raw data of ChIP-sequencing generated from this study have been deposited to the GEO database under the accession codes - GSE240957 [https://www.ncbi.nlm.nih.gov/geo/query/acc.cgi?acc=GSE240957]. Original data underlying this manuscript can be accessed from the Stowers Original Data Repository at http://www.stowers.org/research/publications/libpb-2418. Source data are provided with this paper.

Research involving human participants, their data, or biological material

Policy information about studies with human participants or human data. See also policy information about sex, gender (identity/presentation), and sexual orientation and race, ethnicity and racism.

Reporting on sex and gender	N/A
Reporting on race, ethnicity, or other socially relevant groupings	N/A
Population characteristics	N/A
Recruitment	N/A
Ethics oversight	N/A

Note that full information on the approval of the study protocol must also be provided in the manuscript.

Field-specific reporting

Please select the one below that is the best fit for your research. If you are not sure, read the appropriate sections before making your selection.

[x] Life sciences   [ ] Behavioural & social sciences   [ ] Ecological, evolutionary & environmental sciences

For a reference copy of the document with all sections, see nature.com/documents/nr-reporting-summary-flat.pdf

Life sciences study design

All studies must disclose on these points even when the disclosure is negative.

Sample size	No prior sample size determination was performed. Sample size was chosen based on our previous experience with visualizing structural features of chromosomes using super-resolution microscopy as published by Potapova et. al., 2019 (https://doi.org/10.1083/jcb.2018101662). The sample size of each experiment is provided in the figure legends in the main manuscript and supplementary information files. Multiple biological replicates were performed for ChIP experiments to ensure reproducibility. 3D-SIM analysis was performed from multiple chromosome spreads (n values have been indicated in figures) from 1-2 biological replicates as stated in figure legends.
Data exclusions	One biological replicate from RPE-1 was unsuccessful yielding very low number of reads and was discarded.
Replication	3 biological replicates were performed for ChIP data from RPE-1 and CHM13. ChIP data from RPE-1 cells are representative of 2 biological replicates. All 3 replicates of ChIP in CHM13 were successful. 1 replicate of ChIP in RPE-1 was unsuccessful. All replicates for 3D SIM imaging were successful.
Randomization	Randomization was not performed. Randomization was not relevant for our study of native centromeres as no comparison between conditions were made.
Blinding	Blinding was not performed as all sequencing dataset for each experiment were processed in parallel. Blinding was not relevant for our study of native centromeres as no comparison between conditions were made.

Reporting for specific materials, systems and methods

We require information from authors about some types of materials, experimental systems and methods used in many studies. Here, indicate whether each material, system or method listed is relevant to your study. If you are not sure if a list item applies to your research, read the appropriate section before selecting a response.

Materials & experimental systems

n/a	Involved in the study
☐	☒ Antibodies
☐	☒ Eukaryotic cell lines
☒	☐ Palaeontology and archaeology
☒	☐ Animals and other organisms
☒	☐ Clinical data
☒	☐ Dual use research of concern
☒	☐ Plants

Methods

n/a	Involved in the study
☐	☒ ChIP-seq
☒	☐ Flow cytometry
☒	☐ MRI-based neuroimaging

Antibodies

Antibodies used

Antibodies used for ChIP are as follows –
1. rabbit anti-RAD21 (Abcam, ab154769, polyclonal) for RPE-1
2. rabbit anti-RAD21 (Abcam, ab992, polyclonal) for CHM13
3. rabbit anti-SA1 (Bethyl, A302-579A, polyclonal)
4. rabbit anti-SA2 (Bethyl, A302-580A, polyclonal)

Antibodies used for microscopy are as follows –
1. rabbit anti-RAD21 (Abcam, ab154769)
2. mouse anti-CENPA (MBL, D115-3, monoclonal clone 3-19)
3. rabbit anti-CENPB (Abcam, ab25734)
4. rabbit anti-GFP (Abcam, ab6556)

Secondary antibodies used –
1. anti rabbit-CF 568 (Biotium, Cat# 20098)
2. anti rabbit-AF 647 (Biotium, Cat# 20047)
3. anti mouse-AF 488 (Biotium, Cat# 20014)

Validation

Rabbit anti-RAD21 (ab154769), rabbit anti-RAD21 (Abcam, ab992), Rabbit anti-SA1 (Bethyl, A302-579A), Rabbit anti-SA2 (Bethyl, A302-580A) were validated by ChIP-PCR at a positive control locus Human (GRCh38/hg38) chr20:1,503,742-1,503,891 and showed band enrichments while no band was seen for an IgG control antibody. The positive control locus was determined from Kojic et al., 2018, Nat Struct Mol Biol 25, 496–504 (2018). https://doi.org/10.1038/s41594-018-0070-4

Rabbit anti-RAD21 (Abcam, ab992) has been previously used for ChIP in many publications including Rao et al., 2017, Cell 171, 305–320. https://doi.org/10.1016/j.cell.2017.09.026

Rabbit anti-SA1 (Bethyl, A302-579A), Rabbit anti-SA2 (Bethyl, A302-580A) have been previously used for ChIP in Viny et. al., 2019, Cell Stem Cell 25(5), 682-696.e8. https://doi.org/10.1016/j.stem.2019.08.003.

Rabbit anti-RAD21 (Abcam, ab154769) has been validated by immunostaining by Abcam [https://www.abcam.com/products/primary-antibodies/rad21-antibody-ab154769.html]

Mouse anti-CENPA (MBL, D115-3, monoclonal clone 3-19) has been validated for immunostaining by MBL [https://www.mblbio.com/bio/g/dtl/A/?pcd=D115-3]

Rabbit anti-CENPB (Abcam, ab25734) has been validated by immunostaining by Abcam [https://www.abcam.com/products/primary-antibodies/cenpb-antibody-ab25734.html]

Rabbit anti-GFP (Abcam, ab6556) has been validated by immunostaining by Abcam [https://www.abcam.com/products/primary-antibodies/gfp-antibody-ab6556.html]

Eukaryotic cell lines

Policy information about cell lines and Sex and Gender in Research

Cell line source(s)

hTERT RPE-1 cells were sourced from ATCC. hTERT CHM13 (CHM13) cells were originally isolated from a hydatidiform mole at Magee-Womens Hospital as part of a research study (IRB MWH-20-054) and were used for completion of the human genome sequence by the T2T-consortium. We obtained the cell line from the T2T-consortium.

Authentication

hTERT CHM13 and hTERT RPE-1 cells were validated in the lab by karyotyping.

Authentication certificate for hTERT RPE-1 cells was obtained from ATCC and states as follows -
Mycoplasma contamination- Not detected
Population doubling capacity - ≥ 15 in complete growth medium

STR profiling:
Amelogenin: X
CSF1PO: 12,14
D13S317: 11,12
D16S539: 11
D5S818: 11
D7S820: 10,11
TH01: 9
TPOX: 8
vWA: 17,18
Penta_D: 9,11
Penta_E: 12,14
D3S1358: 15,16
D21S11: 31,32.2
D18S51: 14,16
D8S1179: 10
FGA: 20,23
D19S433: 14,16.2
D2S1338: 19,24

Mycoplasma contamination

Cells were routinely tested for absence of Mycoplasma contamination using PCR.

Commonly misidentified lines
(See ICLAC register)

None used.

Plants

Seed stocks

Report on the source of all seed stocks or other plant material used. If applicable, state the seed stock centre and catalogue number. If plant specimens were collected from the field, describe the collection location, date and sampling procedures.

Novel plant genotypes

Describe the methods by which all novel plant genotypes were produced. This includes those generated by transgenic approaches, gene editing, chemical/radiation-based mutagenesis and hybridization. For transgenic lines, describe the transformation method, the number of independent lines analyzed and the generation upon which experiments were performed. For gene-edited lines, describe the editor used, the endogenous sequence targeted for editing, the targeting guide RNA sequence (if applicable) and how the editor was applied.

Authentication

Describe any authentication procedures for each seed stock used or novel genotype generated. Describe any experiments used to assess the effect of a mutation and, where applicable, how potential secondary effects (e.g. second site T-DNA insertions, mosiacism, off-target gene editing) were examined.

ChIP-seq

Data deposition

- [x] Confirm that both raw and final processed data have been deposited in a public database such as GEO.
- [x] Confirm that you have deposited or provided access to graph files (e.g. BED files) for the called peaks.

Data access links
May remain private before publication.

The raw sequencing data of ChIP-sequencing generated from this study have been deposited to the GEO database under the accession codes - GSE240957 [https://www.ncbi.nlm.nih.gov/geo/query/acc.cgi?acc=GSE240957]

Files in database submission

RPE-1_Rad21_ip_1_R1.fastq.gz
RPE-1_Rad21_ip_1_R2.fastq.gz
RPE-1_Rad21_ip_2_R1.fastq.gz
RPE-1_Rad21_ip_2_R2.fastq.gz
RPE-1_SA1_ip_1_R1.fastq.gz
RPE-1_SA1_ip_1_R2.fastq.gz
RPE-1_SA1_ip_2_R1.fastq.gz
RPE-1_SA1_ip_2_R2.fastq.gz
RPE-1_SA2_ip_1_R1.fastq.gz
RPE-1_SA2_ip_1_R2.fastq.gz
RPE-1_SA2_ip_2_R1.fastq.gz
RPE-1_SA2_ip_2_R2.fastq.gz
RPE-1_tc_1_R1.fastq.gz
RPE-1_tc_1_R2.fastq.gz
RPE-1_tc_2_R1.fastq.gz
RPE-1_tc_2_R2.fastq.gz
CHM13_Rad21_ip_1_R1.fastq.gz
CHM13_Rad21_ip_1_R2.fastq.gz
CHM13_Rad21_ip_2_R1.fastq.gz

CHM13_Rad21_ip_2_R2.fastq.gz
CHM13_Rad21_ip_3_R1.fastq.gz
CHM13_Rad21_ip_3_R2.fastq.gz
CHM13_SA1_ip_1_R1.fastq.gz
CHM13_SA1_ip_1_R2.fastq.gz
CHM13_SA1_ip_2_R1.fastq.gz
CHM13_SA1_ip_2_R2.fastq.gz
CHM13_SA1_ip_3_R1.fastq.gz
CHM13_SA1_ip_3_R2.fastq.gz
CHM13_SA2_ip_1_R1.fastq.gz
CHM13_SA2_ip_1_R2.fastq.gz
CHM13_SA2_ip_2_R1.fastq.gz
CHM13_SA2_ip_2_R2.fastq.gz
CHM13_SA2_ip_3_R1.fastq.gz
CHM13_SA2_ip_3_R2.fastq.gz
CHM13_tc_1_R1.fastq.gz
CHM13_tc_1_R2.fastq.gz
CHM13_tc_2_R1.fastq.gz
CHM13_tc_2_R2.fastq.gz
CHM13_tc_3_R1.fastq.gz
CHM13_tc_3_R2.fastq.gz
CHM13_Rad21_ref_peaks_chm13.bed
CHM13_Rad21_ref_peaks_hg38.bed
CHM13_SA1_ref_peaks_chm13.bed
CHM13_SA1_ref_peaks_hg38.bed
CHM13_SA2_ref_peaks_chm13.bed
CHM13_SA2_ref_peaks_hg38.bed
RPE-1_Rad21_ref_peaks_chm13.bed
RPE-1_Rad21_ref_peaks_hg38.bed
RPE-1_SA1_ref_peaks_chm13.bed
RPE-1_SA1_ref_peaks_hg38.bed
RPE-1_SA2_ref_peaks_chm13.bed
RPE-1_SA2_ref_peaks_hg38.bed
CHM13_Rad21_ip_avg_rpm_chm13.bw
CHM13_Rad21_ip_avg_rpm_hg38.bw
CHM13_SA1_ip_avg_rpm_chm13.bw
CHM13_SA1_ip_avg_rpm_hg38.bw
CHM13_SA2_ip_avg_rpm_chm13.bw
CHM13_SA2_ip_avg_rpm_hg38.bw
CHM13_tc_avg_rpm_chm13.bw
CHM13_tc_avg_rpm_hg38.bw
RPE-1_Rad21_ip_avg_rpm_chm13.bw
RPE-1_Rad21_ip_avg_rpm_hg38.bw
RPE-1_SA1_ip_avg_rpm_chm13.bw
RPE-1_SA1_ip_avg_rpm_hg38.bw
RPE-1_SA2_ip_avg_rpm_chm13.bw
RPE-1_SA2_ip_avg_rpm_hg38.bw
RPE-1_tc_avg_rpm_chm13.bw
RPE-1_tc_avg_rpm_hg38.bw

Genome browser session
(e.g. UCSC)

CHM13 ChIP-seq data mapped to CHM13 v1.0
https://genome.ucsc.edu/cgi-bin/hgTracks?db=hub_2397929_t2t-chm13-v1.0&lastVirtModeType=default&lastVirtModeExtraState=&virtModeType=default&virtMode=0&nonVirtPosition=&position=chr8%3A41672908%2D48751054&hgsid=1711220994_vSt5Tu862pwEaVJGNdVbMtBQ09nH

RPE-1 ChIP seq data mapped to CHM13 v1.0
http://genome.ucsc.edu/cgi-bin/hgTracks?db=hub_2397929_t2t-chm13-v1.0&lastVirtModeType=default&lastVirtModeExtraState=&virtModeType=default&virtMode=0&nonVirtPosition=&position=chr3%3A86648846%2D88422714&hgsid=1711221808_X5AkJmZTzDmj60LJXJjAtqvAOEWa

Methodology

Replicates

3 biological replicates were performed for ChIP data from RPE-1 and CHM13. One biological replicate from RPE-1 was unsuccessful yielding very low number of reads and was discarded. Hence ChIP data from RPE-1 cells are representative of 2 biological replicates. All 3 replicates of ChIP in CHM13 were successful and are in agreement.

Sequencing depth

Cell line Sample Total number of reads Coverage Length of reads (bp) Paired-end or single-end Uniquely mapped reads
CHM13 CHM13_RAD21_1 37181544 2.98 150 Paired-end 31748036

CHM13 CHM13_RAD21_2 10202822 0.72 150 Paired-end 7693453
CHM13 CHM13_RAD21_3 37471953 2.99 150 Paired-end 31873737
CHM13 CHM13_SA1_1 40874847 3.13 150 Paired-end 33361788
CHM13 CHM13_SA1_2 40085046 3.08 150 Paired-end 32825257
CHM13 CHM13_SA1_3 39709932 3.07 150 Paired-end 32701703
CHM13 CHM13_SA2_1 38194394 2.92 150 Paired-end 31136897
CHM13 CHM13_SA2_2 37814082 2.77 150 Paired-end 29559914
CHM13 CHM13_SA2_3 38687587 2.96 150 Paired-end 31522549
CHM13 CHM13_INPUT_1 25092672 1.83 150 Paired-end 19567861
CHM13 CHM13_INPUT_2 33409694 2.53 150 Paired-end 26994342
CHM13 CHM13_INPUT_3 38550422 2.90 150 Paired-end 30970664
RPE1 RPE1_RAD21_1 25512390 2.00 150 Paired-end 21325539
RPE1 RPE1_RAD21_2 18676606 1.39 150 Paired-end 14779282
RPE1 RPE1_SA1_1 26150346 1.88 150 Paired-end 20039272
RPE1 RPE1_SA1_2 23623835 1.80 150 Paired-end 19223273
RPE1 RPE1_SA2_1 22531593 1.70 150 Paired-end 18106222
RPE1 RPE1_SA2_2 17386693 1.32 150 Paired-end 14035204
RPE1 RPE1_INPUT_1 26555913 1.91 150 Paired-end 20329836
RPE1 RPE1_INPUT_2 24826794 1.53 150 Paired-end 16342805

Antibodies

Antibodies used for ChIP are as follows – rabbit anti-RAD21 (Abcam, ab154769) for RPE-1, rabbit anti-RAD21 (Abcam, ab992) for CHM13, rabbit anti-SA1 (Bethyl, A302-579A), rabbit anti-SA2 (Bethyl, A302-580A).

Peak calling parameters

ChIP-seq data was aligned using bwa with the following parameters: bwa mem -k 50 -c 1000000. Secondary and supplementary alignments were excluded using samtools to filter out reads with SAM FLAG 2308. Peaks were called using macs2 with the following parameters: -g 2.9e9 -q 0.01. RNA Seq data were aligned to the CHM13 genome using STAR with default parameters.

Data quality

All reference peaks called by MACS passed FDR 5%.

Software

Illumina Real Time Analysis version RTA 2.4.11 and bcl2fastq2 v2.20 were run to demultiplex reads and generate FASTQ files.
